# Site-selective fatty acid chain conjugation of the N-terminus of the recombinant human granulocyte colony-stimulating factor

**DOI:** 10.3389/fbioe.2024.1360506

**Published:** 2024-03-18

**Authors:** Xu-Dong Wang, Zhi-Hao Su, Jie Du, Wei-Jia Yu, Wen-Long Sun

**Affiliations:** ^1^ College of Pharmaceutical Science, Zhejiang University of Technology, Hangzhou, China; ^2^ Institute of Biomedical Research, School of Life Sciences, Shandong University of Technology, Zibo, China

**Keywords:** recombinant human granulocyte colony-stimulating factor, rhG-CSF, fatty acid chain conjugation, site-selective modification, long-acting rhG-CSF, serum half-life

## Abstract

The clinical application of the recombinant human granulocyte colony-stimulating factor (rhG-CSF) is restricted by its short serum half-life. Herein, site-selective modification of the N-terminus of rhG-CSF with PAL-PEG_3_-Ph-CHO was used to develop a long-acting rhG-CSF. The optimized conditions for rhG-CSF modification with PAL-PEG_3_-Ph-CHO were: reaction solvent system of 3% (w/v) Tween 20 and 30 mM NaCNBH_3_ in acetate buffer (20 mmol/L, pH 5.0), molar ratio of PAL-PEG_3_-Ph-CHO to rhG-CSF of 6:1, temperature of 20°C, and reaction time of 12 h, consequently, achieving a PAL-PEG_3_-Ph-rhG-CSF product yield of 70.8%. The reaction mixture was purified via preparative liquid chromatography, yielding the single-modified product PAL-PEG_3_-Ph-rhG-CSF with a HPLC purity exceeding 95%. The molecular weight of PAL-PEG_3_-Ph-rhG-CSF was 19297 Da by MALDI-TOF-MS, which was consistent with the theoretical value. The circular dichroism analysis revealed no significant change in its secondary structure compared to unmodified rhG-CSF. The PAL-PEG_3_-Ph-rhG-CSF retained 82.0% of the *in vitro* biological activity of unmodified rhG-CSF. The pharmacokinetic analyses showed that the serum half-life of PAL-PEG_3_-Ph-rhG-CSF was 7.404 ± 0.777 h in mice, 4.08 times longer than unmodified rhG-CSF. Additionally, a single subcutaneous dose of PAL-PEG_3_-Ph-rhG-CSF presented comparable *in vivo* efficacy to multiple doses of rhG-CSF. This study demonstrated an efficacious strategy for developing long-acting rhG-CSF drug candidates.

## Introduction

The recombinant human granulocyte colony-stimulating factor (rhG-CSF) has 175 amino acids with an approximate molecular weight of 18.8 kDa. It is commonly produced using *Escherichia coli* and is clinically used to prevent and treat symptoms such as leukopenia (Liongue et al., 2009) and bone marrow dysfunction caused by tumor radiation therapy or chemotherapy (Morstyn et al., 2001). However, rhG-CSF has a short half-life (only 1–2 h in the human body) and requires repeated administration, which can increase the difficulty of treatment and reduce patient compliance. Hence, developing a long-acting protein strategy is imperative.

Currently, common strategies for protein modification include fusion proteins, formulation changes, and PEGylation. Although PEGylation (Yang et al., 2004) is the most widely used long-acting strategy, difficulties in body degradation can lead to PEG accumulation and potential immune reactions when exogenous macromolecules enter the body (Verhoef and Anchordoquy, 2013). For example, after intravenous injection of PEGylated L-asparaginase, 46% of patients developed anti-PEG antibodies during treatment, accelerating clearance during subsequent injections (Armstrong et al., 2007). Therefore, new, longer-acting protein modification strategies are urgent.

In recent years, fatty acid chain modification has been recognized as a novel, long-acting strategy due to its ability to bind to protein and peptide drugs covalently. Fatty acid chain modification presents several advantages: 1) The fatty acid chain can reversibly bind to human serum albumin (HSA), the most abundant protein in extracellular fluid, with an approximate molecular weight of 66 kDa and a half-life of 19 days in the human body (Sleep et al., 2013). After fatty acid chain modification, the drug can form a complex with HSA (Bhattacharya et al., 2000), increasing the molecular size and extending the half-life through a neonatal Fc receptor (FcRn)-mediated mechanism (Chaudhury et al., 2003). 2) Fatty acid chains are small molecules with little steric hindrance, and their binding to the original protein does not decrease its activity (Lim et al., 2013). 3) Fatty acid chains have good biocompatibility and do not induce immunogenic responses in the body (Schultz et al., 2018). 4) Fatty acid chain modification can increase the liposolubility of protein and peptide drugs, enhancing cell membrane permeability and facilitating transmembrane absorption (Lau et al., 2015).

Moreover, fatty acid chain modification has been successfully applied for long-acting protein and peptide drug formulations, such as Exendin-4 (Sun et al., 2016), HGA (Human Growth Hormone) (Ramírez-Andersen et al., 2018), and Interferon-α2 (Balliu and Baltzer, 2017). Several fatty acid chain-modified drugs have been successfully marketed, such as Levemir (Beji et al., 2020), Tresiba (Atkin et al., 2015), Victoza (Sjöholm, 2010), Ozempie (Christou et al., 2019), Sogroya (Juul Kildemoes et al., 2021). Typically, the modification sites of proteins include *ε*-amine of lysine residues, *α*-amine of N-terminus, and thiol group of cysteine residues (Roberts et al., 2002; Rosen and Francis, 2017). Moreover, the site-specific modification strategy is more preferred in recent years. Our previous study demonstrated that site-specific fatty acid chain modification at the thiol group of Cys18 in rhG-CSF could extend its serum half-life (Wang et al., 2022). Besides, rhG-CSF has four lysine residues (Lys17, Lys24, Lys35, and Lys41), making them unsuitable for site-specific modification. Alternatively, a potential modification site is the N-terminal amino acid residue, which is unique in single-chain peptides (Rosen and Francis, 2017). The pKa of α-NH_2_ at the N-terminus typically falls below 7, while the pKa of ε-NH_2_ of lysine ranges from 10.0 to 10.2. Hence, controlling the pH allows site-selective modification at the N-terminus (Hu and Sebald, 2011).

**FIGURE 1:**
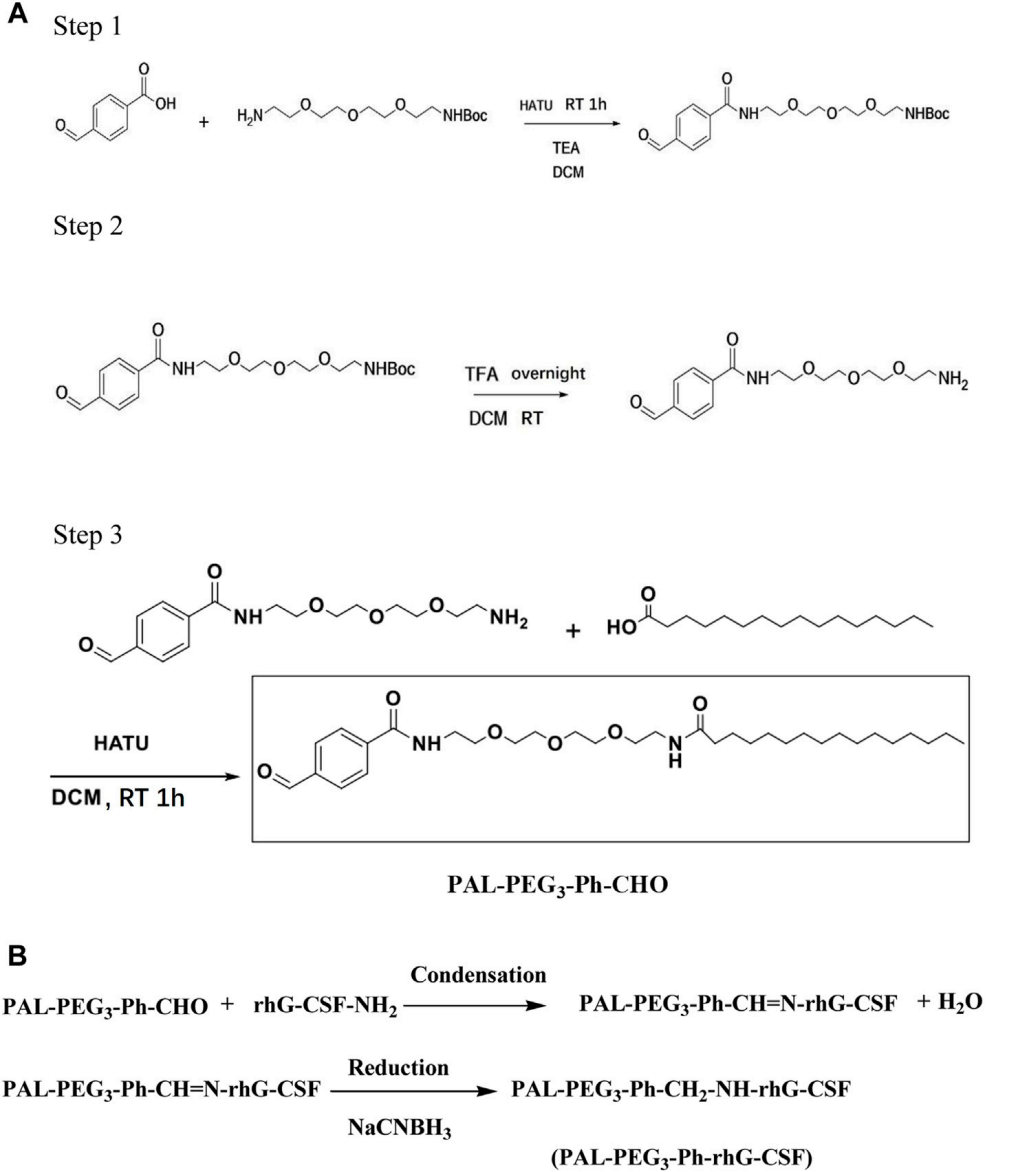
Synthesis routes of PAL-PEG_3_-Ph-CHO **(A)** and PAL-PEG_3_-Ph-rhG-CSF **(B)**.

Herein, we developed a novel fatty acid chain modification strategy for specific conjugation at the N-terminal amino acid residue of rhG-CSF using a novel palmitoyl-based modifier, PAL-PEG_3_-Ph-CHO (Figure 1). Palmitoyl-based modifiers, which have high HSA affinity (Liu et al., 2023), are commonly used to extend the serum half-life of proteins and peptides. We used PAL-PEG_3_-Ph-CHO to modify rhG-CSF in an acetic acid buffer and optimized the reaction conditions to maximize the reaction yield. We identified key factors and optimized process parameters in the corresponding system, including pH, temperature, molar ratio of PAL-PEG_3_-Ph-CHO to rhG-CSF and reaction time. Finally, we conducted a preliminary evaluation of the fatty acid chain-modified rhG-CSF product to provide foundational data for future drug development.

## Materials and methods

### Materials

The rhG-CSF was obtained from Lunan Pharmaceutical Group Co., Ltd., with a purity of at least 98% by liquid chromatography. Tert-butyloxycarbonyl-polyethylene glycol-amine was purchased from Xi’an Point Chemical Technology Co., Ltd. The SDS-PAGE reagent kit and Bradford protein quantification test kit were purchased from Shanghai Sangon Biotech Co., Ltd. All other reagents used in this study were of analytical grade and were acquired from China National Pharmaceutical Group Chemical Reagent Co., Ltd.

### Animals

Male Kunming mice (16–20 g) were obtained from the Experimental Animal Center of Shandong (Jinan, China); the license number is SCXK 2014-0007. The animal husbandry system was maintained under standard laboratory conditions. All animal procedures were performed following the Guidelines for the Care and Use of Laboratory Animals of Shandong University of Technology and were approved by the Animal Ethics Committee of Shandong University of Technology.

### Synthesis of PAL-PEG_3_-Ph-CHO

The synthesis route of PAL-PEG_3_-Ph-CHO is presented in Figure 1A. In the first step, 1.0 g of p-formylbenzoic acid was dissolved in dichloromethane and combined with 1.5 equivalents of hexafluorophosphate aza-benzotriazole tetramethyl uronium (HATU). After 10 min, 1.1 equivalents of Boc-protected t-butylcarbonyl-polyethylene glycol-amino and 1.5 equivalents of triethylamine were added. The reaction was allowed to proceed for 1 hour; thin layer chromatography (TLC) was used to monitor the reaction. Upon completion, the organic phase was diluted with additional dichloromethane, followed by sequential washing with water and diluted hydrochloric acid. The resulting mixture was dried and evaporated to dryness, yielding an oily organic product of 2.8 g.

In the second step, the product obtained from the first step was dissolved in 10 mL of dichloromethane and exposed to trifluoroacetic acid overnight for Boc deprotection. The reaction was monitored via TLC until the starting material disappeared. The resultant yellow oil was dried and weighed, yielding 2.0 g.

In the third step, the product from the second step was dissolved in dichloromethane and combined with 1.0 equivalent of HATU, 1.0 equivalent of palmitic acid, and 1.5 equivalents of triethylamine. The reaction was monitored using TLC and allowed to proceed for 1 hour. Upon completion, the organic phase was diluted with dichloromethane, washed with water and diluted hydrochloric acid, dried, and evaporated to yield a white solid. The product, PAL-PEG_3_-Ph-CHO, was purified via column chromatography using pure ethyl acetate (EA). The column chromatography conditions were as follows: The sample (white solid powder 1.5 g) was subjected to column chromatography on a silica gel column (2.4 × 100 cm, Kieselgel 60, Merck, Germany) and then equilibrated with hexane. Elution was performed sequentially with 750 mL of pure ethyl acetate. Each elution fraction (50 mL) was collected and assessed using RP-HPLC analysis to determine the desired product. Finally, the desired fractions were combined and recrystallized using a mixture of dichloromethane and ether to obtain the PAL-PEG_3_-Ph-CHO product with HPLC purity exceeding 95%.

The collected fractions from column chromatography and the recrystallized product were assessed using RP-HPLC analysis as follows: XBridge BEH Shield RP18 column (2.5 µm, 2.1 mm × 50 m), flow rate of 1.20 mL/min, and detection at 254 nm. The mobile phase consisted of 0.01% formic acid in water (mobile phase A) and 0.01% formic acid in acetonitrile (mobile phase B). The elution gradient was set as 0–2.65 min, 50%–100% B; 2.65–2.70 min, 100%–2% B; then maintained for 3 min before the detection was halted.

Subsequently, the chemical structure of the obtained product was confirmed via the characteristic peaks in the Fourier-transform infrared spectroscopy (Supplementary Figure S1). The molecular weight was determined via mass spectrometry (Supplementary Figure S1). The chemical shift of each proton in PAL-PEG_3_-Ph-CHO was determined by 1H Nuclear magnetic resonance (NMR) to determine if the chemical structure matches the designed structure. The sample was dissolved in deuterated chloroform, and data were recorded using a 500 MHz NMR spectrometer.

### Conjugation of rhG-CSF with PAL-PEG_3_-Ph-CHO

rhG-CSF was pre-dissolved in 20 mmol/L acetate buffer at investigated pH. PAL-PEG_3_-Ph-CHO was pre-dissolved in 20 mmol/L acetate buffer at the same investigated pH containing Tween 20 and NaCNBH_3_. The two solutions were mixed to form a reaction solution system at a final volume of 10 mL, a rhG-CSF concentration of 1 mg/mL and a NaCNBH_3_ concentration of 30 mM. First, the reaction conditions, including Tween 20 concentration, buffer pH, temperature, and molar ratio of PAL-PEG_3_-Ph-CHO to rhG-CSF, were optimized at reaction time of 10 h. The Tween 20 concentration was 1, 2, 3, 4, 5, 6, or 7% (w/v). The pH of acetate buffer was 4.0, 4.5, 5.0, 5.5, or 6.0. The reaction temperature was 4, 20, 30, or 40 °C. The molar ratio of PAL-PEG_3_-Ph-CHO to rhG-CSF was 4:1, 5:1, 6:1, 7:1, or 8:1. The standard condition for the analysis of each factor was as follows: Tween 20 concentration of 5% (w/v) and 30 mM NaCNBH_3_ in acetate buffer (20 mmol/L, pH 4.5), mole ratio of PAL-PEG_3_-Ph-CHO to rhG-CSF of 5:1, pH 4.5, temperature 20°C, and reaction time 10 h. Subsequently, the reaction time (0.5, 1, 2, 5, 6, 7, 9, 11, 12, 13, 24 h) was investigated under the optimized reaction conditions of the above-mentioned four factors.

To identifying the interactions among these factors, orthogonal experiments were conducted at three levels for each of the four factors (Tween 20 concentration, pH, temperature and the molar ratio of PAL-PEG_3_-Ph-CHO to rhG-CSF) using an L9 orthogonal table (Supplementary Tables S1, S2).

Each sample (450 μL) was taken at different times and mixed with 50 μL of a 2 mol/L glycine solution to terminate the reaction, and then, immediately analyzed by RP-HPLC to calculate the concentration of the desired product (PAL-PEG_3_-Ph-rhG-CSF) based on the peak area. To evaluate the reaction efficiency, the yield of PAL-PEG_3_-Ph-rhG-CSF was calculated by Eq. 1.

$$\text{Yield of PAL}-{\text{PEG}}_{3}-\text{Ph}-\text{rhG}-\text{CSF }\left(\%\right)=\frac{\text{PAL}-{\text{PEG}}_{3}-\text{Ph}-\text{rhG}-\text{CSF}\;\left(\text{mol}\right)}{\text{The}\;\text{initial}\;\text{rhG}-\text{CSF}\;\left(\text{mol}\right)}\times 100$$ (1)

### Purification of the PAL-PEG_3_-Ph-rhG-CSF

After the modification reaction, the mixture was firstly dialyzed against distilled water using a dialysis bag (MWCO of 3.0 kDa) for 24 h to remove the Tween 20, and further purified using a Sepax Bio-C8 column (21.2 × 250 mm, 10 μm, 300Å, Sepax Technologies Inc., United States) on a Shimadzu SPD-M20 A system (Shimadzu, Japan) to obtain PAL-PEG_3_-Ph-rhG-CSF. The purification process involved a gradient elution method with a flow rate of 10.0 mL/min and a detection wavelength of 280 nm. The mobile phase consisted of 0.01% trifluoroacetic acid (TFA) in water (A) and 0.01% TFA in acetonitrile (B). The elution conditions were as follows: 0–5 min, 40%–60% B; 5–30 min, 60%–70% B, and data collection was stopped at 30 min. The injection volume was 500 μL. The eluted peak was collected, dialyzed, and exchanged into a 20 mmol/L acetate-sodium acetate buffer (pH 4.2). The final product was stored at 4°C for subsequent studies.

### Mass spectroscopy (MS) analysis

The molecular weights of PAL-PEG_3_-Ph-CHO, rhG-CSF and fatty acid chain-modified rhG-CSF products were determined by MS analysis. More details were shown in Supplementary Figures S1–S3 **.**

### Circular dichroism (CD) spectroscopy analysis

The CD spectroscopy was used to assess the secondary structure of PAL-PEG_3_-Ph-rhG-CSF compared to unmodified rhG-CSF, which served as the control. The experiment was conducted on a CD-spectrometer (J-810, Jasco, Japan) using a 20 mM acetate buffer at pH 4.2 with a rhG-CSF equivalent concentration of 0.2 mg/mL for both PAL-PEG_3_-Ph-rhG-CSF and rhG-CSF. The cuvette path length was 1 cm. Measurements were recorded at 25°C and the wavelength ranges were 200–260 nm with a scanning step of 0.2 nm and a scanning speed of 100 nm/min. Each spectrum was scanned five times and the average spectrum was recorded.

### *In vitro* bioactivity assay

To evaluate the *in vitro* biological activity of PAL-PEG_3_-Ph-rhG-CSF, a 3-(4,5-dimethylthiazol-2-yl)-2,5-diphenyltetrazolium bromide (MTT) assay was conducted to assess its effects on the proliferation of NFS60 cells (Behi et al., 2018). Unmodified rhG-CSF was the control. Briefly, NFS-60 cells were cultured in RPMI 1640 medium supplemented with 12.5% horse serum and 2.5% fetal bovine serum. The cell density was adjusted to 2.0 × 10^5^ cells/mL. Cells were incubated at 37°C in a humidified atmosphere with 95% air and 5% CO_2_. Next, 50 μL of the adjusted cells were added to duplicate wells of a 96-well plate. The rhG-CSF and PAL-PEG_3_-Ph-rhG-CSF were adjusted at a rhG-CSF equivalent concentration of 0.03 μg/mL using the NFS-60 cells’ culture medium and then added to the wells. The plate was incubated at 37°C and 5% CO_2_ for 48 h. Then, 20 µL of MTT solution was added to each well, followed by incubation under the same conditions for 4 h. Subsequently, 100 µL of SDS solution (25%) was added to each well, and the plate was incubated at room temperature overnight to dissolve the formazan crystals formed. Finally, the absorbance at 570 nm was measured using a microplate reader (Bio-Rad, model 550, United States).

### RP-HPLC analysis

The reaction mixtures derived from the modification of rhG-CSF with PAL-PEG_3_-Ph-CHO and the purified PAL-PEG_3_-Ph-rhG-CSF were analyzed using a Sepax Bio-C8 column (4.6 mm × 250 mm, 5 μm, 300Å, Sepax Technologies Inc., United States) on a Shimadzu SPD-M20 A system (Shimadzu, Japan) at 25°C. The analysis was performed using a gradient elution method with a flow rate of 1.0 mL/min and a detection wavelength of 280 nm. The mobile phase consisted of 0.01% TFA in water (A) and 0.01% TFA in acetonitrile (B). The elution conditions were as follows: 0–5 min, 40%–55% B; 5–30 min, 55%–80% B, and data collection was stopped at 30 min. The injection volume was 20 μL.

### *In vivo* pharmacokinetic properties in mice

Normal mice were randomly divided into groups and sampled as specified in Supplementary Table S3 after subcutaneous administration. Blood samples were collected from the retro-orbital sinus of six animals at each designated time, as listed in Supplementary Table S3. After blood collection, mice were euthanized by cervical dislocation. The serum was separated by centrifugation at a specific speed for 30 min and stored at −20°C until further analysis. The concentrations of rhG-CSF or PAL-PEG_3_-Ph-rhG-CSF in the blood samples were determined using the Human G-CSF DuoSet assay kit (purchased from R&D Systems, United States) and a Model 450 microplate reader (Bio-RAD, United States) with a 96-well enzyme-linked immunosorbent assay (ELISA) plate, following the manufacturer’s instructions in the Human G-CSF DuoSet assay kit manual.

### *In vivo* efficacy study in mice

Except for the normal control group, mice were injected with cyclophosphamide (CTX) at a dose of 1 mg/kg body weight for three consecutive days. Five normal and model mice were randomly selected to measure white blood cell (WBC) counts to verify the success of the model establishment.

Next, successfully modeled mice were divided into six groups (*n* = 12): normal control, model control, high-dose rhG-CSF (0.2 mg/kg), low-dose rhG-CSF (0.1 mg/kg), high-dose PAL-PEG_3_-Ph-rhG-CSF (1.0 mg/kg), and low-dose PAL-PEG_3_-Ph-rhG-CSF (0.5 mg/kg) groups. Specific administration protocols are shown in Supplementary Table S4.

The normal control and model control groups were subcutaneously injected with physiological saline on days 1–5. After administration, mice were euthanized on day 6 by cervical dislocation, and blood was collected. Three blood samples were taken from each mouse, and WBC counts were recorded using a hemocytometer.

### Statistical analysis

Statistical analyses were performed using GraphPad Prism 6 software, and Students’ t-tests were used to determine significant differences between groups. The values of *p < 0.05,**p < 0.01, and ***p < 0.001 were considered significant for intergroup comparisons.

## Results and discussion

### Synthesis and characterization of PAL-PEG_3_-Ph-CHO

A novel PAL-PEG_3_-Ph-CHO was synthesized (Figure 1) for site-selective conjugation at the N-terminal amino acid residue of rhG-CSF. The purity of the recrystallized product (Supplementary Figures S1A) was 98% by HPLC. Then, we characterized the structure of PAL-PEG_3_-Ph-CHO using infrared spectrum, MALDI-TOF-MS and 1H NMR (Supplementary Figures S1B–D). As shown in Supplementary Figure S1B **,** the peaks at 850 cm^−1^ indicated the presence of a disubstituted benzene structure, while the peak at 790 cm^-1^ suggested the presence of ether bond stretching vibrations. Peaks at 1,547 cm^−1^ and 3,298 cm^-1^ corresponded to N-H bending and stretching vibrations, respectively, which is consistent with the theoretical structure. As shown in Supplementary Figure S1C, the molecular weight of PAL-PEG_3_-Ph-CHO was 562 Da, which is consistent with the theoretical value. As shown in Supplementary Figure S1D; Supplementary Table S5, the theoretical structure was also confirmed by 1H NMR. These findings confirmed that the obtained product was the target compound, PAL-PEG_3_-Ph-CHO, with high purity, suitable for further rhG-CSF modification.

### Modification of rhG-CSF with PAL-PEG_3_-Ph-CHO

**FIGURE 2:**
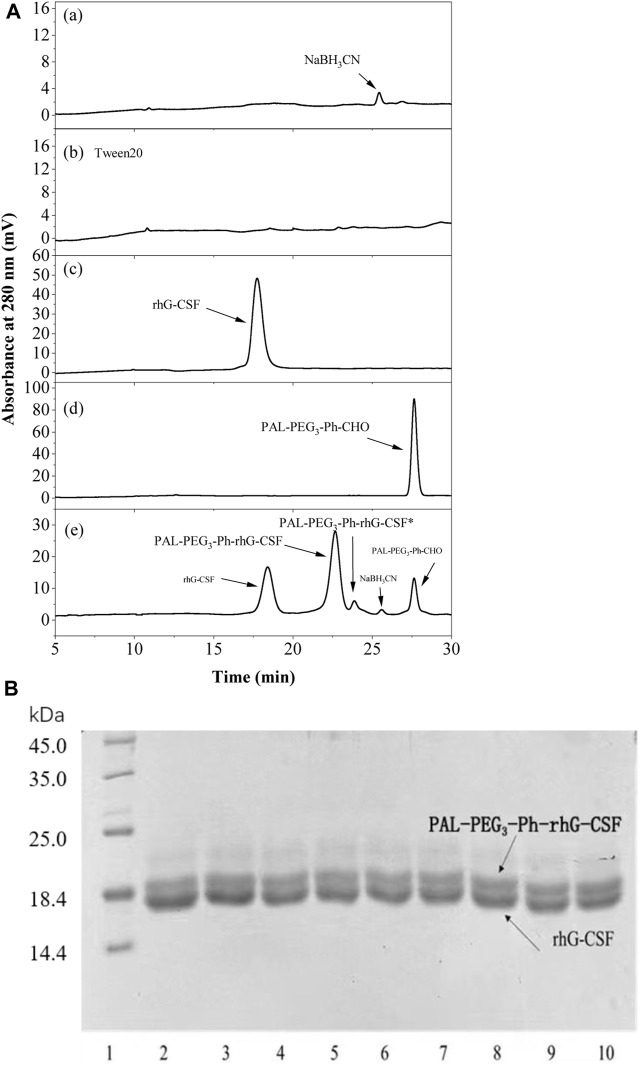
RP-HPLC analysis **(A)** and SDS-PAGE analysis **(B)** of the reaction mixture of the modification of rhG-CSF with PAL-PEG_3_-Ph-CHO. For RP-HPLC analysis **(A)**, (a) NaCNBH_3_, (b) Tween 20, (c) rhG-CSF, (d) PAL-PEG_3_-Ph-CHO, and (e) Reaction mixture obtained at 10 h. For SDS-PAGE analysis **(B)**, lane 1: Protein maker, lane 2-9: the reaction mixture respectively obtained at 4–12 h. The bands corresponded to rhG-CSF and PAL-PEG_3_-Ph-rhG-CSF could be identified based on the result in Figure 4B. The other reaction conditions: rhG-CSF (1 mg/mL) and PAL-PEG_3_-Ph-CHO (mole ratio of PAL-PEG_3_-Ph-CHO to rhG-CSF 5:1) were reacted in acetate buffer (20 mmol/L, pH 4.5) containing 3% (w/v) Tween 20 and 30 mM NaCNBH_3_ at 20°C.

First, the RP-HPLC analysis and SDS-PAGE analysis methods were establish to effectively evaluate the modification effect. As shown in Figures 2Ae, the peaks of NaCNBH_3_, rhG-CSF, and PAL-PEG_3_-Ph-CHO were identified according to their retention times by comparison with the corresponding standards (Figures 2Aa, c, d). Two unknown elution peaks at retention times of 22.8 and 23.8 min, respectively, were collected and detected by MS (Supplementary Figures S2, S3), which indicated that both the peaks corresponded to single-fatty acid chain-modified rhG-CSF. Previous studies demonstrated that aldehyde chemistry was preferential and high-selective modification at *α*-amine of N-terminus, and concurrently minor competitive modification at *ε*-amine of lysine residues under acidic condition (Wang et al., 2011; Wang et al., 2018). Therefore, the peak at retention time of 22.8 min was identified as single-fatty acid chain-modified rhG-CSF at *α*-amine of N-terminus, which was named as PAL-PEG_3_-Ph-rhG-CSF. Meanwhile, the peak at retention time of 23.8 min was identified as single-fatty acid chain-modified rhG-CSF at ε-amine of lysine residues, which was named as PAL-PEG_3_-Ph-rhG-CSF*. Besides, the multi-fatty acid chain-modified rhG-CSF products were undetected. The reaction mixture was further analyzed by SDS-PAGE. As shown in Figure 2B; Supplementary Figure S4, two clear protein bands were observed in the reaction mixture. Since the molecular weight of the single-fatty acid chain-modified rhG-CSF was slightly higher than that of unmodified rhG-CSF, the bands corresponded to rhG-CSF and PAL-PEG_3_-Ph-rhG-CSF were slightly separated. Similar results were also observed in previous study (Cho et al., 2020). As the reaction time increased from 4 to 10 h, the upper band became darker while the lower band became lighter, further confirming the generation of PAL-PEG_3_-Ph-rhG-CSF. Besides, as shown in Figure 2Ae, the peaks of rhG-CSF, PAL-PEG_3_-Ph-CHO, NaCNBH_3_, PAL-PEG_3_-Ph-rhG-CSF and PAL-PEG_3_-Ph-rhG-CSF* were effectively separated, indicating the established RP-HPLC analysis method could be used to quantitatively determined the content of these components.

**FIGURE 3:**
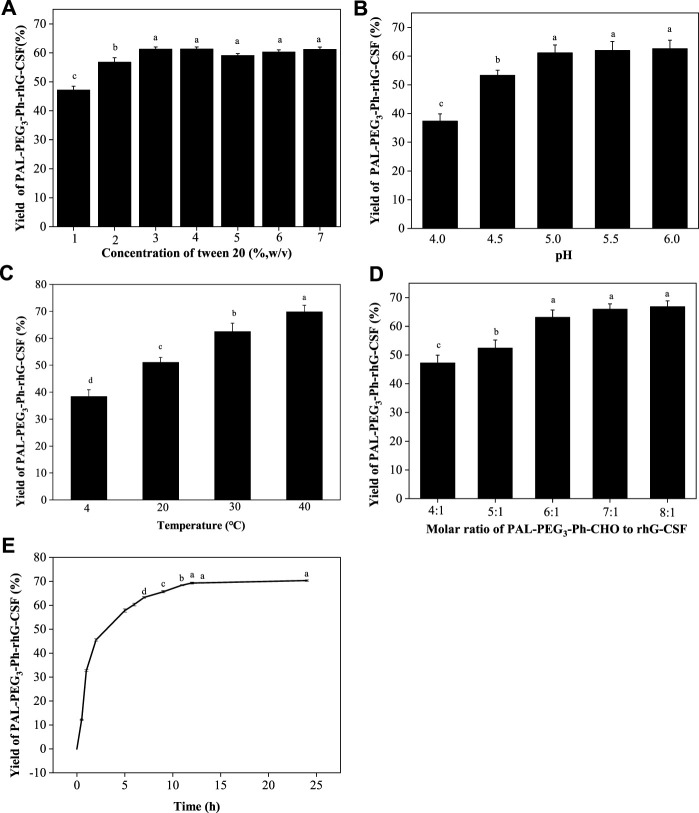
Effects of Tween 20 concentration **(A)**, pH **(B)**, temperature **(C)** and the molar ratio of PAL-PEG_3_-Ph-CHO to rhG-CSF **(D)** and the reaction time **(E)** on the conjugation of rhG-CSF with PAL-PEG_3_-Ph-CHO. The standard condition for the analysis of each of the four parameters (Tween 20 concentrations, pH, temperature and the molar ratios of PAL-PEG_3_-Ph-CHO to rhG-CSF) was as follows: Tween 20 concentration of 5% (w/v) and 30 mM NaCNBH_3_ in acetate buffer (20 mmol/L, pH 4.5), mole ratio of PAL-PEG_3_-Ph-CHO to rhG-CSF of 5:1, pH 4.5, temperature 20°C and reaction time 10 h. The effect of reaction time was investigated at reaction solvent system of 3% (w/v) Tween 20 and 30 mM NaCNBH_3_ in acetate buffer (20 mmol/L, pH 5.0), molar ratio of PAL-PEG_3_-Ph-CHO to rhG-CSF of 6:1, temperature of 20°C. Data were expressed as mean ± SD (*n* = 3). Values with different letters (a, b, c and d) were significantly statistically different from each other (one-way ANOVA followed by Tukey’s multiple comparison, *p* < 0 .05). Values without significant difference in each comparison share the same letter.

Subsequently, the factors affecting the modification reaction were optimized to obtain a high yield PAL-PEG_3_-Ph-rhG-CSF product. Since PAL-PEG_3_-Ph-CHO is high hydrophobic, while rhG-CSF is highly hydrophilic. Therefore, establishing a proper reaction solution system to promote their contact is the key to promote the reaction. In this study, Tween 20, which has been proved as solubilizer for poorly water-soluble drugs dissolving into aqueous solution (Desai and Park, 2004) and stabilizer for PEGylated rhG-CSF (Piedmonte and Treuheit, 2008), was used as a cosolvent to enhance the solubility of PAL-PEG_3_-Ph-CHO into aqueous solution, thus improving reaction efficiency. As shown in Figure 3A, the yield of PAL-PEG_3_-Ph-rhG-CSF increased with increasing Tween 20 concentration and reached a plateau at 3% (w/v). There were no significant differences observed between concentration 3%–7% (w/v) of Tween 20. Therefore, 3% (w/v) Tween 20 was selected as the optimized condition for subsequent experiments. Site-selective modification of the N-terminus of proteins usually occurred under slightly acidic conditions (Hu and Sebald, 2011). Thus, pH 4.0, 4.5, 5.0, 5.5, and 6.0 were investigated. The highest yield was achieved at pH 5.0 and the reaction yield between pH 5.0 ∼ pH 6.0 showed no significant difference (Figure 3B). Commercially available rhG-CSF injections are typically stored in acetic acid buffer at a pH of around 4.2, and the isoelectric point of rhG-CSF is 6.1 (Liu et al., 2020). Besides, the ratio of competitive modification at ε-amine of lysine residues increased with the increasing pH (Wang et al., 2011; Wang et al., 2018). Hence, pH 5.0 was selected as the optimized condition for subsequent experiments. The reaction temperature, molar ratio of PAL-PEG_3_-Ph-CHO to rhG-CSF, and reaction time are also crucial factors affecting the modification reaction. Herein, we conducted the reaction overnight at 4, 20, 30, and 40°C. The reaction yield increased with increasing temperature (Figure 3C). However, extended exposure to high temperature could result in protein inactivation due to the prolonged reaction time. Thus, we selected 20°C as the optimized reaction temperature. As shown in Figure 3D, the molar ratio of PAL-PEG_3_-Ph-CHO to rhG-CSF gradually increased the reaction yield. When the molar ratio reached 6:1, further increasing the molar ratio did not significantly increase the reaction yield. Besides, multi-fatty acid chain-modified rhG-CSF products might generate with the increasing molar ratio (Wang et al., 2011; Wang et al., 2018). Therefore, we selected 6:1 as the optimized molar ratio of PAL-PEG_3_-Ph-CHO to rhG-CSF. Under the above-mentioned optimized reaction conditions, the reaction yield reached a plateau at approximately 12 h (Figure 3E). Thus, 12 h was selected as the optimized reaction time for subsequent experiments. In summary, a high PAL-PEG_3_-Ph-rhG-CSF yield (70.8%) was obtained under the above-mentioned optimized reaction conditions at solvent system of 3% (w/v) Tween 20 in acetate buffer (20 mmol/L, pH 5.0), molar ratio of PAL-PEG_3_-Ph-CHO to rhG-CSF of 6:1, temperature of 20°C, and reaction time of 12 h.

The effects of the four factors (Tween 20 concentration, pH, temperature and the molar ratio of PAL-PEG_3_-Ph-CHO to rhG-CSF) and their interactions effects on the reaction yield of PAL-PEG3-Ph-rhG-CSF were further investigated using orthogonal experiments. As shown in Supplementary Table S2; Supplementary Figure S5, the parameters affecting the reaction were ranked in the following order: temperature > pH > molar ratio of PAL-PEG_3_-Ph-CHO to rhG-CSF > Tween 20 concentration. There was a direct interaction between temperature and pH, while there was no significant interaction between molar ratio of PAL-PEG_3_-Ph-CHO to rhG-CSF and Tween 20 concentration. We also carried out the validation experiment under the optimized reaction conditions (Tween 20 concentration 4% (w/v), pH 6.0, temperature 30°C and the molar ratio of PAL-PEG_3_-Ph-CHO to rhG-CSF 7:1) derived from orthogonal experiments. Consequently, a PAL-PEG_3_-Ph-rhG-CSF product yield of 72.3% was achieved, which was slightly higher than that of the above-mentioned single factor optimization experiment (yield of 70.8%). It is worth noting that the optimized reaction conditions derived from orthogonal experiments were all the experimental maximum boundary conditions (Supplementary Table S2; Supplementary Figure S5), thus only local optimization result was obtained. To obtain the global optimization result and more details on the interaction effects of different factors, the combined optimization strategy using response surface methodology and kinetic analysis (Wang et al., 2018) should be taken into consideration in further studies.

Considering the cost of raw materials, the effect of pH on the site-selective modification at α-amine of N-terminus and the effect of temperature on the deactivation of rhG-CSF, we still selected the single factor optimized reaction conditions for subsequent experiments.

### Purification of PAL-PEG_3_-Ph-rhG-CSF

**FIGURE 4:**
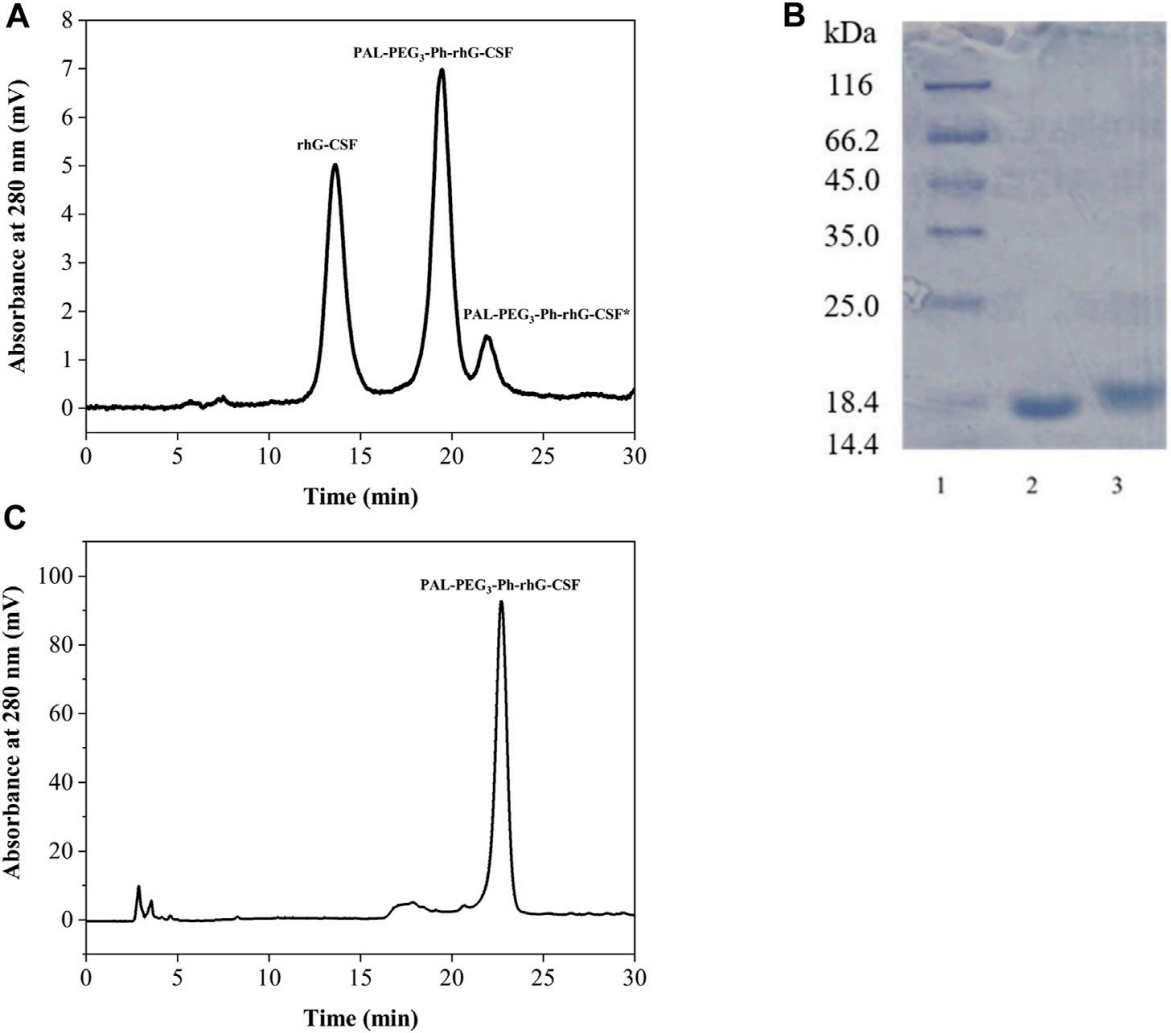
**(A)** Purification of reaction mixture of the modification of rhG-CSF with PAL-PEG_3_-Ph-CHO by preparative liquid chromatography; **(B)** SDS-PAGE analysis of rhG-CSF and purified PAL-PEG_3_-Ph-rhG-CSF.Lane 1: protein marker, lane 2: rhG-CSF, Lane 3: purified PAL-PEG_3_-Ph-rhG-CSF; **(C)** Purity analysis of PAL-rhG-CSF by RP-HPLC.

The reaction mixture obtained from the optimized conditions was effectively separated and purified using preparative liquid chromatography (Figure 4A). Two major elution peaks were collected and analyzed by the RP-HPLC analysis and SDS-PAGE analysis. The collected fractions underwent post-processing steps, including acetonitrile removal, dialysis, and concentration, to obtain a highly purified PAL-PEG_3_-Ph-rhG-CSF. As shown in Figures 4B, C, the purified PAL-PEG_3_-Ph-rhG-CSF had both high electrophoresis and HPLC purity (both >95%) and could be used for subsequent characterization.

### Characterization of PAL-PEG_3_-Ph-rhG-CSF

**FIGURE 5:**
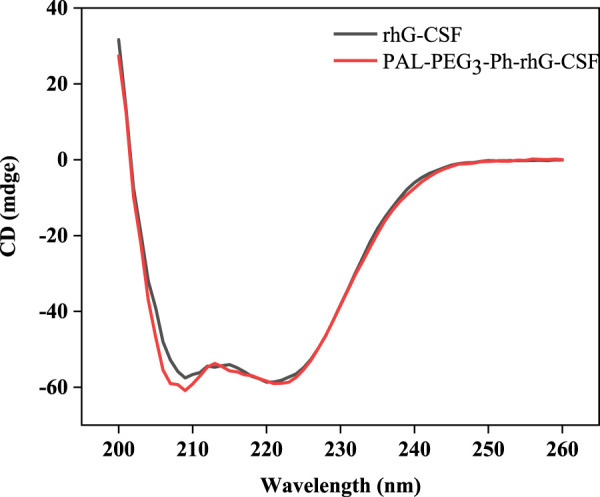
Circular dichroism (CD) of rhG-CSF and PAL-PEG_3_-Ph-rhG-CSF.

**FIGURE 6:**
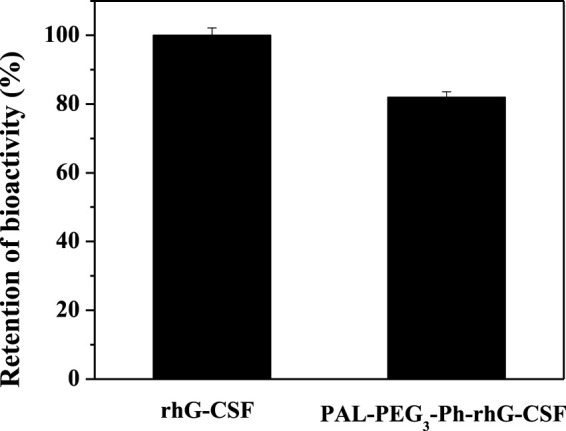
*In vitro* bioactivities of rhG-CSF and PAL-PEG_3_-Ph-rhG-CSF.

The molecular weight of the rhG-CSF was 18819 Da. In contrast, the molecular weight of PAL-PEG_3_-Ph-rhG-CSF was 19297 Da (Supplementary Figures S1A, B). This result indicated that only one fatty acid chain was conjugated into rhG-CSF. Additionally, the measured molecular weight of the isolated product is consistent with the expected value. This result confirmed the successful synthesis of the desired product PAL-PEG_3_-Ph-rhG-CSF. Circular dichroism (CD) was used to analyze the structure of PAL-PEG_3_-Ph-rhG-CSF compared to unmodified rhG-CSF (Figure 5). PAL-PEG_3_-Ph-rhG-CSF and rhG-CSF had similar far-UV CD spectra, indicating that the fatty acid chain modification had little effect on the structure of rhG-CSF. The double negative peaks observed around 200–220 nm in the CD spectra represent the α-helix structure in the protein. The first peak was associated with the π-π* electronic transition, while the second peak was attributed to the n-π* electronic transition. The negative peak at 210 nm was characteristic of β-sheet conformation. Overall, these CD spectra indicated that both proteins contained α-helix and β-sheet structures. After the modification with PAL-PEG_3_-Ph-CHO, the biological activity of rhG-CSF remained 82.0% (Figure 6), which was slightly lower than C15- rhG-CSF (87.2%) derived from thiol-group modification at the Cys18 of rhG-CSF in our previous study (Wang et al., 2022), while higher than mono-PEG_10k_-rhG-CSF(42.7%) derived PEGylation at the N-terminus of rhG-CSF with 10 kDa mPEG-ALD in previous study (Behi et al., 2018).

**FIGURE 7:**
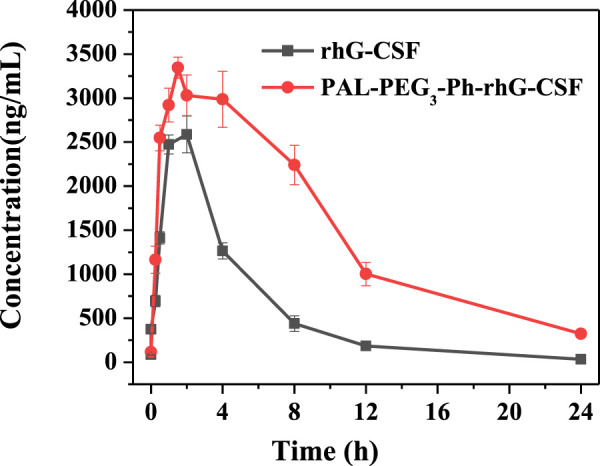
Pharmacokinetic analysis of rhG-CSF and PAL-PEG_3_-Ph-rhG-CSF.

As shown in Figure 7; Table 1, the subcutaneous administration of the drug in mice resulted in a half-life of 1.816 ± 0.471 h for rhG-CSF. Meanwhile, after the modification (PAL-PEG_3_-Ph-rhG-CSF), the half-life was extended to 7.404 ± 0.777 h, approximately 4.08 times longer than the unmodified rhG-CSF. Besides, the half-life of PAL-PEG_3_-Ph-rhG-CSF was higher than that of C15- rhG-CSF (5.72 h) (Wang et al., 2022) and PEG-rhG-CSF(7.05 h) (Tanaka et al., 1991) in previous studies.

**TABLE 1:** Pharmacokinetic parameters of rhG-CSF and PAL-PEG_3_-Ph-rhG-CSF.

Parameter^a^	rhG-CSF	PAL-PEG_3_-Ph-rhG-CSF
T_1/2_/h	1.816 ± 0.471	7.404 ± 0.777
AUC(0-∞)/ng·h·mL^-1^	12632.270 ± 499.468	41362.154 ± 2213.824
AUC(0-t)/ng·h·mL^-1^	12614.586 ± 489.943	37384.948 ± 1503.660
C_max_/ng·mL^-1^	2728.729 ± 202.806	3348.617 ± 119.680
T_peak_(h)	1.667 ± 0.471	1.583 ± 0.186
Lag time/h	0.143 ± 0.025	0.070 ± 0.073
CL/f (s) (L·h^-1^·kg^-1^)	0.079 ± 0.003	0.024 ± 0.001
V/f (c) (L·kg^-1^)	0.206 ± 0.048	0.258 ± 0.020

^a^ Note: T_1/2_, Serum half-life; AUC_(0-t)_, Area under drug concentration *versus* time curve; C_max_, Maximal drug concentration; T_peak_, Time of maximal drug concentration; CL/f (s), Clearance over bioavailability; V/f (c), Apparent volume of distribution.

Additionally, the area under the curve (AUC) (0-∞) of PAL-PEG_3_-Ph-rhG-CSF was 41362.154 ± 2213.824 ng·h·mL^-1^, which was 3.27 times higher than rhG-CSF (Table 1), indicating that PAL-PEG_3_-Ph-rhG-CSF exhibited higher bioavailability compared to rhG-CSF. Besides, the AUC (0-t) of PAL-PEG_3_-Ph-rhG-CSF (37384.948 ± 1,503.660 ng·h·mL^-1^) was higher than that of C15-rhG-CSF (24,322.13 ± 1,075.57 ng·h·mL^-1^) in our previous study (Wang et al., 2022). The results indicated that PAL-PEG_3_-Ph-rhG-CSF had a better bioavailability than that of C15-rhG-CSF.

**FIGURE 8:**
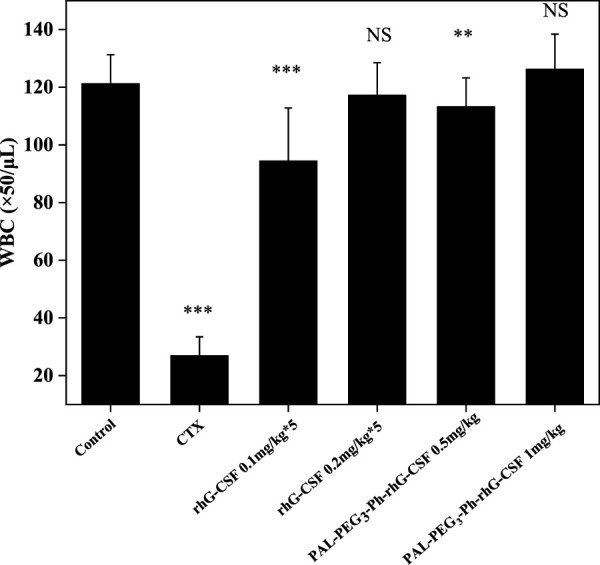
Effects of rhG-CSF and PAL-PEG_3_-Ph-rhG-CSF on the WBC counts in CTX treated mice. PAL-PEG_3_-Ph-rhG-CSF are single injections; rhG-CSF is divided into 5 days, 0.1 mg/kg or 0.2 mg/kg per day, a total of 0.5 mg/kg or 1.0 mg/kg. The values of *p < 0.05, **p < 0.01 and ***p < 0.001 were considered significant for intergroup comparisons with the normal control group.

The mice’s WBC count significantly decreased after CTX treatment, also indicating the successful establishment of the model (Figure 8). Subcutaneous injection of rhG-CSF and PAL-PEG_3_-Ph-rhG-CSF in CTX-treated mice increased the peripheral blood WBC count. For low-dose groups (0.5 mg/kg), although the WBC counts of both the multiple doses rhG-CSF group and single-dose PAL-PEG_3_-Ph-rhG-CSF group did not fully recover to the normal level, the WBC count of the single-dose PAL-PEG_3_-Ph-rhG-CSF group was significant higher than the single-dose rhG-CSF group, indicating that PAL-PEG_3_-Ph-rhG-CSF by single-dose administration had better *in vivo* efficacy than rhG-CSF by multiple doses administration at the same total dose of 0.5 mg/kg. For high-dose groups (1.0 mg/kg), the WBC counts of both the multiple doses rhG-CSF group and single-dose PAL-PEG_3_-Ph-rhG-CSF group could fully recover to the normal level, and showed no significant differences, indicating an equivalent *in vivo* efficacy between PAL-PEG_3_-Ph-rhG-CSF by single-dose administration and rhG-CSF by multiple doses administration at the same total dose of 1.0 mg/kg. Besides, the *in vivo* efficacy of PAL-PEG_3_-Ph-rhG-CSF was equivalent to that of C15-hG-CSF in our previous study (Wang et al., 2022). These results indicated the potential of PAL-PEG_3_-Ph-rhG-CSF as a drug candidate. To deeply evaluate the potential of PAL-PEG_3_-Ph-rhG-CSF to be developed as a candidate drug, it is *in vivo* pharmacodynamics and safety (e.g., toxicology and immunogenicity) should be investigated in future studies.

## Conclusion

In this study, we successfully designed and synthesized a novel fatty acid chain modifier, PAL-PEG_3_-Ph-CHO, which can specifically modify the N-terminus of rhG-CSF in an acetic acid buffer solution. The structure of PAL-PEG_3_-Ph-CHO was confirmed using infrared spectrum, mass spectrometry and 1H NMR. Due to the extreme hydrophobicity of the modifier and the hydrophilicity of the peptide, we chose Tween 20 to improve the solubility of PAL-PEG_3_-Ph-CHO into the aqueous solution. We optimized various parameters, including Tween 20 concentration, reaction pH, temperature, molar ratio of PAL-PEG_3_-Ph-CHO to rhG-CSF, and reaction time. After optimization, the final reaction conditions were: reaction solvent system of 3% (w/v) Tween 20 and 30 mM NaCNBH_3_ in acetate buffer (20 mmol/L, pH 5.0), molar ratio of PAL-PEG_3_-Ph-CHO to rhG-CSF of 6:1, temperature of 20°C, and reaction time of 12 h, consequently, achieving a PAL-PEG_3_-Ph-rhG-CSF product yield of 70.8%. After purification using preparative liquid chromatography, the target product PAL-PEG_3_-Ph-rhG-CSF with the HPLC purity exceeding 95% was obtained. Additionally, the MALDI-TOF-MS demonstrated the molecular weight of PAL-PEG_3_-Ph-rhG-CSF was 19297 Da. The CD analysis demonstrated that the structure of PAL-PEG_3_-Ph-rhG-CSF did not undergo significant changes compared to unmodified rhG-CSF. The NFS-60 cells test demonstrated that PAL-PEG_3_-Ph-rhG-CSF remained 82.0% of the unmodified G-CSF. Finally, the pharmacokinetic *in vivo* pharmacokinetic properties in mice showed that the half-life of PAL-PEG_3_-Ph-rhG-CSF was 7.404 ± 0.777 h, 4.08 times longer than rhG-CSF (1.816 ± 0.471 h), indicating a significant extension of half-life. Besides, PAL-PEG_3_-Ph-rhG-CSF achieved equivalent *in vivo* efficacy than compared to rhG-CSF. These results demonstrated the potential of PAL-PEG_3_-Ph-rhG-CSF as a novel candidate drug and an efficacious strategy for developing long-acting protein and peptide drugs.

## Data Availability

The original contributions presented in the study are included in the article/Supplementary Material, further inquiries can be directed to the corresponding authors.
