# Supplementary material for: Site-selective fatty acid chain conjugation of the N-terminus of the recombinant human granulocyte colony-stimulating factor
Source: Front Bioeng Biotechnol. 2024 Mar 18;12:1360506. doi: 10.3389/fbioe.2024.1360506 (PMC10993259; doi:10.3389/fbioe.2024.1360506)
Supplement: Supplementary file 1 [file DataSheet1.docx]

**Supplementary Table S1. The** **factors and levels of orthogonal experiments**

| Level | Factors | | | |
| --- | --- | --- | --- | --- |
|  | Tween20 concentration (%,w/v) | pH | Temperature (℃) | Molar ratio of PAL-PEG_3_-Ph-CHO to rhG-CSF |
| 1 | 2 | 4.0 | 10 | 5:1 |
| 2 | 3 | 5.0 | 20 | 6:1 |
| 3 | 4 | 6.0 | 30 | 7:1 |

**Supplementary Table S2. Orthogonal experimental combination and results**

| Test No. | Tween20 concentration (%, w/v) | pH | Temperature (℃) | Molar ratio of PAL-PEG_3_-Ph-CHO to rhG-CSF | Yield |
| --- | --- | --- | --- | --- | --- |
| 1 | 1 | 1 | 1 | 1 | 57.08 |
| 2 | 1 | 2 | 2 | 2 | 67.94 |
| 3 | 1 | 3 | 3 | 3 | 71.89 |
| 4 | 2 | 1 | 2 | 3 | 68.23 |
| 5 | 2 | 2 | 3 | 1 | 69.21 |
| 6 | 2 | 3 | 1 | 2 | 66.92 |
| 7 | 3 | 1 | 3 | 2 | 68.43 |
| 8 | 3 | 2 | 1 | 3 | 67.89 |
| 9 | 3 | 3 | 2 | 1 | 69.68 |
| k1 | 65.637 | 64.580 | 63.963 | 65.323 |  |
| k2 | 68.120 | 68.347 | 68.617 | 67.763 |  |
| k3 | 68.667 | 69.497 | 69.843 | 69.337 |  |
| R | 3.030 | 4.917 | 5.880 | 4.014 |  |

**Supplementary Table S3.** Method of subcutaneous administration and sampling of hG-CSF and PAL-PEG_3_-Ph-rhG-CSF for the *in vivo* pharmacokinetic study.

| Sampe | Mice (n) | Dose (mg/kg) | Sampling time (h) |
| --- | --- | --- | --- |
| rhG-CSF | 60 | 1.0 | 0, 0.08, 0.25, 0.5, 1, 2, 4, 8, 12, 24 |
| PAL-PEG_3_-Ph-rhG-CSF | 60 | 1.0 | 0, 0.25, 0.5, 1, 1.5, 2, 4, 8, 12, 24 |

**Supplementary Table S4.** Method of subcutaneous administration of rhG-CSF and PAL-PEG_3_-Ph-rhG-CSF for the *in vivo* efficacy study.

| Group | Mice (n) | Dose (mg/kg) | Administrationtime (day) |
| --- | --- | --- | --- |
| Normal control group | 12 | - | D1、D2、D3、D4、D5 |
| Model control group | 12 | - | D1、D2、D3、D4、D5 |
| Low-dose rhG-CSF | 12 | 0.1 *5 | D1、D2、D3、D4、D5 |
| High-dose rhG-CSF | 12 | 0.2 *5 | D1、D2、D3、D4、D5 |
| Low-dose PAL-PEG_3_-Ph-rhG-CSF | 12 | 0.5 | D1 |
| High-dose  PAL-PEG_3_-Ph-rhG-CSF | 12 | 1.0 | D1 |

**Note:** “-”expressed that the mice in normal control group and model control group were subcutaneously injected withequal volume of saline.

“D1, 2,3,…”expressed that the injection was carried out at the first, second, and third,…day.

**Supplementary Table S5. ^1^H-NMR data of PAL-PEG_3_-Ph-CHO**

| Chemical shift | Chemical group |
| --- | --- |
| 0.8 | -CH_3_- |
| 1.25 | -CH_2_- |
| 7.9 |  |
| 10.0 | -CHO |

(A)


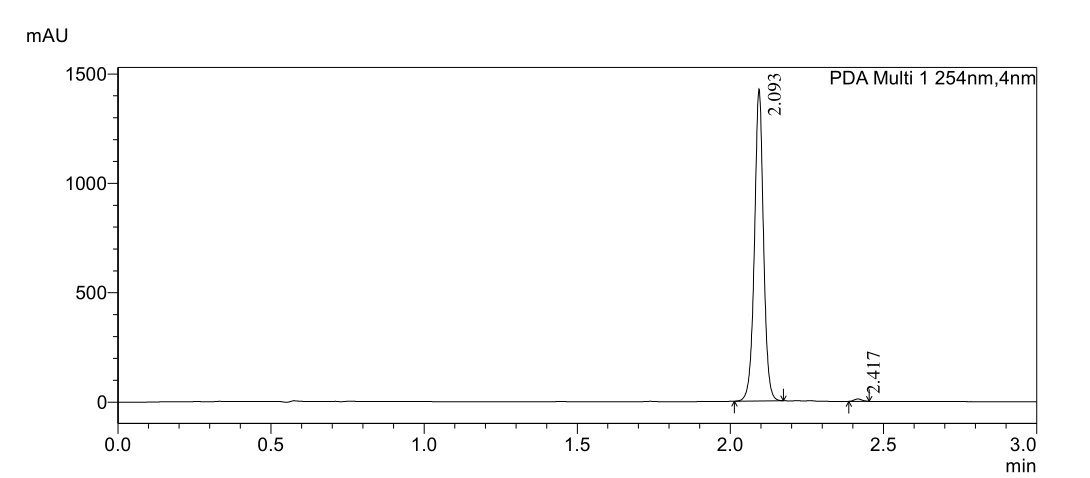


(B)

(C)

[M+Na]+

[M+Na+CH3OH]+

[M+H]+

(D)


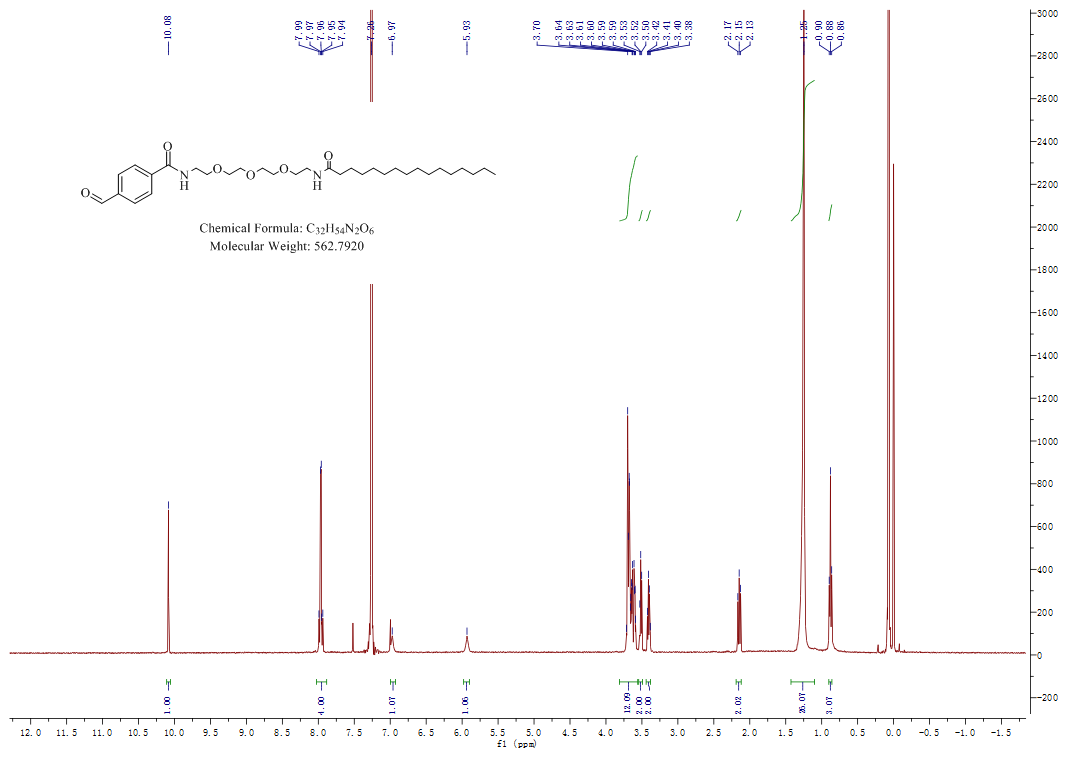


**Supplementary Figure S1.** RP-HPLC analysis (A), Infrared spectrum (B), Mass spectrum(C) and Hydrogen spectrum (D) of PAL-PEG_3_-Ph-CHO.

Infrared spectrum (B) analysis was conducted on a Thermo Nicolet 6700 Fourier transform infrared spectrometer (Thermo Fisher Scientific Inc., Waltham, USA). PAL-PEG3-Ph-CHO powder (1 mg) was added to 100 mg of KBr. The mixture was uniformly compressed and detected. Sample spectra were collected as the average of at least 64 scans each with a resolution of 0.5 cm^−1^.The experiment was repeated three times.

Mass spectrum (C) were acquired in reflection mode between m/z 100 and 1000 on a 5800 Proteomics Analyzer (Applied Biosystems, Framingham, MA, USA) with the Nd: YAG laser at 355 nm, a repetition rate of 200 Hz and an acceleration voltage of 20 kV. The matrix solution was a saturated solution of sinapinic acid in methanol: water (50:50, v:v) containing 0.1% trifluoroacetic acid (TFA).

In Fig.S1(C), the peak 563.4069, 585.3886 and 617.4153 Da corresponded to [M+H]^+^, [M+Na]^+^ and [M+Na+CH_3_OH]^+^, respectively.

(A)

**
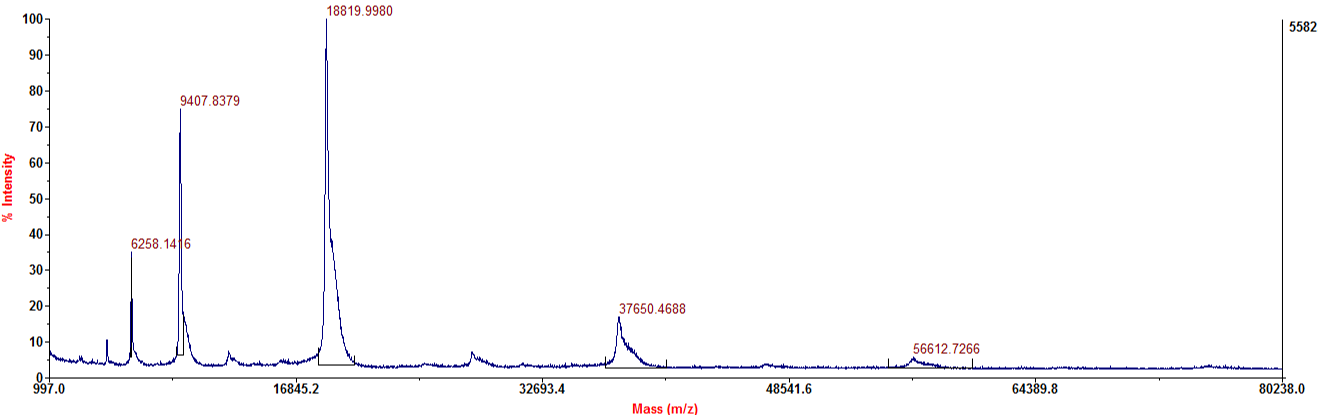
**

[M+3H]^3^+

[M+2H]^2^+

[M+H]+

[3M+H]+

[2M+H]+

(B)

[M+H]+


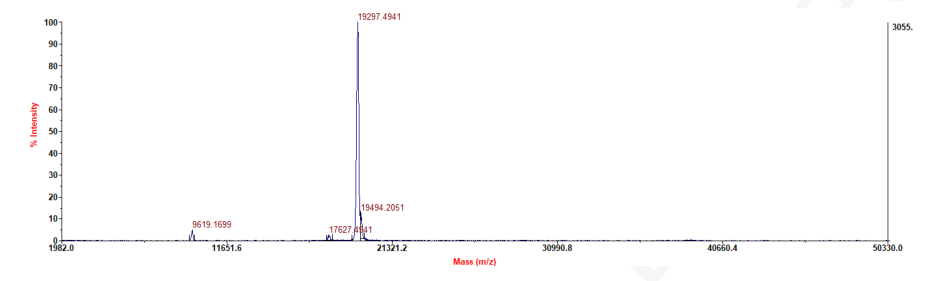


[M+2H]^2^+

**Supplementary Figure S2.** MALDI-TOF MS analysis of rhG-CSF(A),PAL-PEG_3_-Ph-rhG-CSF(B).

The analysis was carried out on 4800 proteomics Analyzer (Applied Biosystems, Foster City, CA, USA) equipped with a 337 nm nitrogen laser. The matrix solution was a saturated solution of sinapinic acid in acetonitrile : water (50:50, v:v) containing 0.1% trifluoroacetic acid (TFA). The mass spectra data were obtained in a linear and positive-ion mode at an acceleration voltage of 20 kV.

In Fig.S2 (A), the peak 6258.1416, 9407.8370, 18819.9980, 37650.4688 and 56612.7266 Da corresponded to [M+3H]^3^+ [M+2H]^2^+, [M+H]+, [2M+H]^+^ and [3M+H]^+^, respectively. That is, the observed average molecular weight of rhG-CSF was 18819 Da. In Fig.S2 (B), the peak 9619.1699 and 19297.4941 Da correspond to [M+2H]^2^+ and [M+H]+, respectively. That is, the observed average molecular weight of PAL-PEG_3_-Ph-rhG-CSF was 19296 Da.


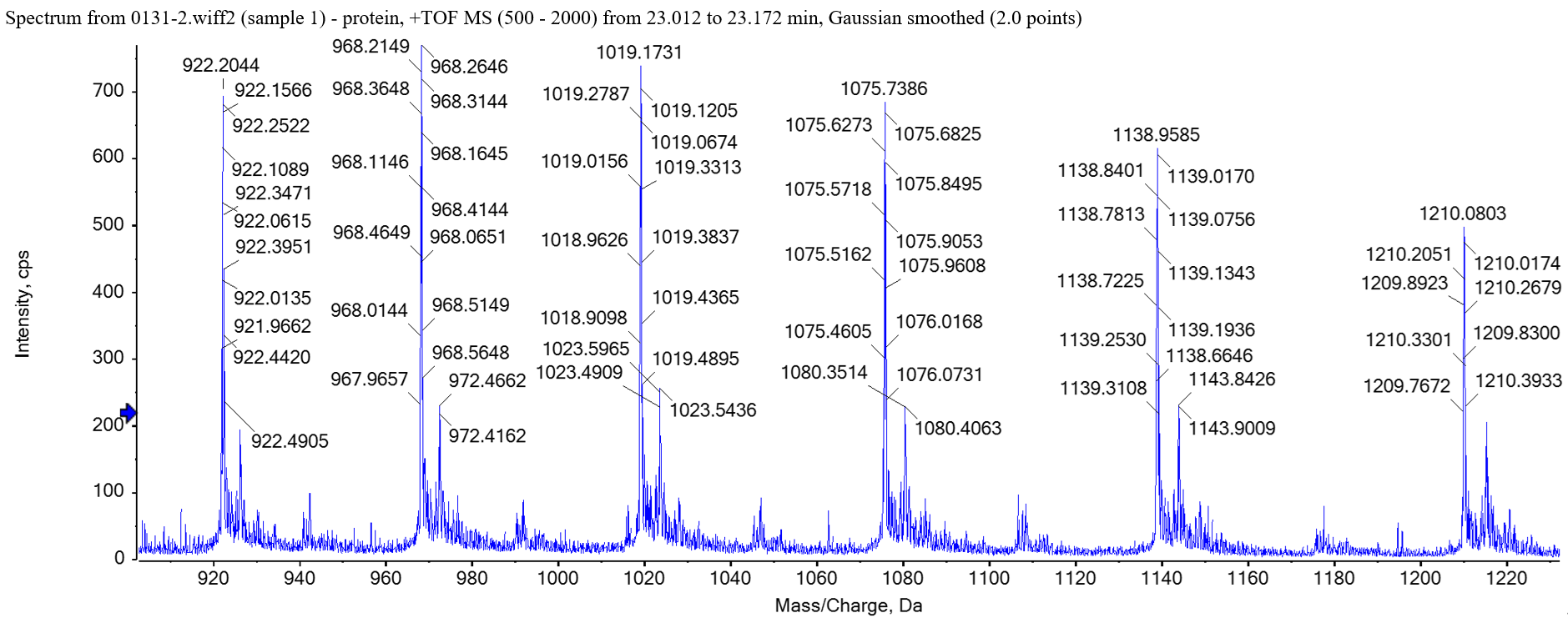


[M+20H]20+

[M+21H]21+

[M+19H]19+

[M+18H]18+

[M+17H]17+

[M+16H]16+

**Supplementary Figure S3.** ESI-QTOF MS analysis of the peak (PAL-PEG_3_-Ph-rhG-CSF*, RT=23.8 min) in Figure 2A(e).

The analysis was performed on an AB SCIEX X500R QTOFMS (AB Sciex, Concord, ON, Canada)   with a Turbo V source (AB Sciex, Concord, ON, Canada) operated with [electrospray ionization](https://www.sciencedirect.com/topics/chemistry/electrospray-ionization) (ESI) probe. ESI-QTOF MS conditions were as follows: The desolvation gas (nitrogen) was heated to 400 °C and delivered at a flowrate of 10 L/min. Ionization was achieved in the positive ion mode at source voltage of 5.5 kV. Nitrogen was used as the nebulizer gas with nebulizer pressure of 25 psi. Scan range was 500-2000 m/z.

The peak 922.2044, 968.2646, 1019.1731, 1075.7386, 1138.9585 and 1210.0803 Da corresponded to[M+21H]^21^+ [M+20H]^20^+, [M+19H]^19^+, [2M+18H]^18+^, [M+17H]^17+^ and [M+16H]^16^+, respectively. That is, the observed average molecular weight of PAL-PEG_3_-Ph-rhG-CSF* was 19345 Da.

**Supplementary Figure S4.** Densitometry analysis of the lanes corresponded to PAL-PEG_3_-Ph-rhG-CSF and rhG-CSF in Figure 2B.

**Supplementary Figure S5.** Visualization plot of interaction effects.
